# Effect of Ionic Liquids with Different Structures on Rheological Properties of Water-Based Drilling Fluids and Mechanism Research at Ultra-High Temperatures

**DOI:** 10.3390/molecules29174206

**Published:** 2024-09-05

**Authors:** Haoxian Shi, Yanjiang Yu, Yingsheng Wang, Zijie Ning, Zhihua Luo

**Affiliations:** 1 Guangzhou Marine Geological Survey, Guangzhou 510075, China; shihaoxian00@163.com (H.S.); yuyanjiang2004@163.com (Y.Y.); gmgs-wys@163.com (Y.W.); ningzijie@outlook.com (Z.N.); 2 School of Engineering and Technology, China University of Geosciences, Beijing 100083, China

**Keywords:** ionic liquids, water-based drilling fluids, rheological property, ultra-high temperature, mechanism analysis

## Abstract

The rheology control of water-based drilling fluids at ultra-high temperatures has been one of the major challenges in deep or ultra-deep resource exploration. In this paper, the effects of 1-ethyl-3-methylimidazolium bis(trifluoromethanesulfonimide) (ILA), 1-ethyl-3-methylimidazolium tetrafluoroborate (ILB) and N-methyl, butylpyrrolidinium bis(trifluoromethanesulfonimide) (ILC) on the rheological properties and filtration loss of polymer-based slurries at ultra-high temperatures (200 °C and 240 °C) are investigated by the American Petroleum Institute (API) standards. The results show that ionic liquids with different structures could improve the high-temperature rheological properties of polymer-based drilling fluids. The rheological parameter value (YP/PV) of the polymer-based slurry formulated with ILC is slightly higher than that with ILA at the same concentration, while the YP/PV value of the polymer-based slurry with ILA is slightly higher than that with ILB, which is consistent with the TGA thermal stability of ILA, ILB, and ILC; the thermal stability of ILC with pyrrolidine cations is higher than that of ILA with imidazole cations, and the thermal stability of ILA with bis(trifluorosulfonyl)amide anions is higher than that of ILB with tetrafluoroborate anions. Cation interlayer exchange between organic cation and sodium montmorillonite can improve the rheological properties of water-based drilling fluids. And meantime, the S=O bond in bis(trifluorosulfonyl)amide ions and the hydroxyl group of sodium montmorillonite may form hydrogen bonds, which also may increase the rheological properties of water-based drilling fluids. ILA, ILB, and ILC cannot reduce the filtration loss of polymer-based drilling fluids at ultra-high temperatures.

## 1. Introduction

In recent years, the rapid development of the economy has increased the demand for energy consumption. The field of resource exploration and exploitation, such as oil/gas and geothermal resources, has shifted from shallow and medium depths to deep and ultra-deep depths [1,2,3,4]. The international petroleum industry defines a formation temperature of 204 °C as ultra-high temperature [5]. High-temperature-resistant water-based drilling fluids are one of the key technologies for drilling these resources [6]. Water-based drilling fluid is a multi-phase dispersed system in which clay is dispersed in the continuous water phase, and its performance, such as rheological property, filtration loss, inhibition and lubrication, is adjusted by adding various additives. Among them, rheological property is the most important property of drilling fluids and a key factor for drilling quickly and safely, which directly affects drilling fluids’ ability to carry drilling cuttings and clean boreholes and affects the design of hydraulic parameters [7,8]. Under high-temperature conditions, agglomeration or dispersion of the clay particles causes the viscosity of the drilling fluid to decrease or increase, which results in losing the functions of carrying cuttings and cleaning boreholes. In the meantime, the amount of filtration loss of drilling fluids under high temperatures increases greatly, which also causes the well wall to become unstable and damages the reservoir. The factors mentioned above are macroscopically manifested as the rheological properties are out of control under high temperatures [9].

Currently, water-soluble polymers are commonly added to control and adjust the rheological properties of water-based drilling fluids under high temperatures. The main polymers include 2-acrylamido-2-methylpropanesulfonate (AMPS) and acrylic acid (AA), acrylamide (AM) copolymers, sulfonated styrene and maleic anhydride copolymers (SSMA), sulfonated lignite (Resinex) and lignosulfonates, polyanionic cellulose (PAC), sodium carboxymethyl cellulose (NaCMC), and so on [10,11,12,13]. Tehrani et al. synthesized polyvinylpyrrolidone and used it to regulate the high-temperature rheological properties of water-based drilling fluids with temperature resistance up to 180 °C [14]. Li et al. synthesized a tetrapolymer composed of acrylamide (AM), 2-acrylamido-2-methyl-1-propanesulfonic acid (AMPS), 4-styrenesulfonic acid sodium salt (SSS), and N-vinylpyrrolidone (NVP) [15]. Freshwater drilling fluids containing this polymer exhibited good fluid loss control at 200 °C. Huang et al. synthesized a tetrapolymer of acrylic acid (AA), 2-acrylamido-2-methyl-1-propanesulfonic acid (AMPS), maleic anhydride (MA), and dimethyldiallylammonium chloride (DMDAAC) [16]. Water-based drilling fluids containing this polymer still demonstrated good dilution performance at 245 °C.

In recent years, new copolymers, copolymers with different topological structures, inorganic nanomaterials, and the composites of polymers with inorganic nanomaterials have been used to adjust the high-temperature rheological properties of water-based drilling fluids [17,18,19]. Huang et al. prepared polymer-based nanomaterials by incorporating nano-lithium soapstone particles into a terpolymer (AM, AMPS, DMDAAC) [20]. When 2 wt% of this composite was added to water-based drilling fluids, it maintained good rheological stability at 200 °C. Luo et al. synthesized a star-shaped copolymer of AM and AMPS [21]. Compared to linear polymers of the same molecular weight, adding 0.3% of this copolymer to freshwater drilling fluids reduced fluid loss and provided more stable rheological properties at 160 °C. Although the rheological stability of drilling fluids under high temperatures can be enhanced by adding new copolymers or new copolymers with optimized structures, high temperatures reduce the polarity of water and weaken the hydrogen bonds between water molecules, thereby decreasing the solubility of polymers in water [22]. Additionally, these polymers either degrade thermally or cross-link at high temperatures, and most water-soluble polymers fail at temperatures around 200 °C. When temperatures exceed 240 °C, the thermal stability of water-soluble polymers approaches its limit. Therefore, maintaining ideal rheological properties of water-based drilling fluids at ultra-high temperatures is currently a key focus and challenge in this field.

Organic nanoparticles have also been used to regulate the rheological properties of drilling fluids under high temperatures [23,24]. A.R. Ismail et al. studied the rheological properties of freshwater drilling fluids with the addition of multi-walled carbon nanotubes (MWCNTs) at high temperatures [25]. The results showed that this organic nanomaterial could increase the plastic viscosity of drilling fluids from 121 °C to 176 °C and reduce fluid loss. D V. Kosynkin et al. added graphene oxide to water-based drilling fluids [26]. This material effectively reduced fluid loss and improved the rheological properties of the drilling fluids within the temperature range of 140 °C to 210 °C. Other nanoparticles, such as CuO, Fe_2_O_3_, and TiO_2_, have also been proven to effectively improve the rheological properties and fluid loss of drilling fluids [27,28,29], but nanoparticles exhibit poor colloidal stability, and maintaining the colloidal stability requires the addition of high-temperature-resistant surfactants.

Studies of the effects of imidazolium tetrafluoroborate ionic liquids on the rheological properties of water-based drilling fluids at high temperatures have been conducted in our previous research. The research indicated that the ionic liquid at low concentrations could improve the rheological properties of water-based drilling fluids at high temperatures. The imidazolium cations of ILB exchanged with the sodium ions of sodium montmorillonite through cationic interlayer adsorption, thereby improving the high-temperature rheological properties of the drilling fluid [7]. R.A. Khan et al. investigated the effects of ionic liquids with the same 1-allyl-3-methylimidazolium cation but with four different anions (bromine, iodine, chlorine, and dicyanamide) on freshwater-based mud [30]. The results showed that regardless of the anion, the ionic liquids with the same cation could improve the rheological properties of the base mud, but the temperature was below 180 °C. Lili Yang et al. investigated the use of 1-vinyl-3-ethylimidazolium bromide ionic liquid and its corresponding homopolymer as shale hydration inhibitors [31]. The results showed that the base mud containing the ionic liquid exhibited excellent thermal stability, even at temperatures up to 300 °C.

Current research has primarily focused on the effects of ionic liquids with imidazolium cations and tetrafluoroborate or halide anions on the rheological properties of water-based drilling fluids under high temperatures. The literature has reported that the thermal stabilities of 1-ethyl-3-methylimidazolium bis(trifluoromethanesulfonimide) salt (ILA) and N-methyl, butylpyrrolidinium bis(trifluoromethanesulfonimide) salt (ILC) are higher than that of 1-ethyl-3-methylimidazolium tetrafluoroborate (ILB); the latter is thermally decomposed at 330 °C [32,33]. ILA and ILC may be used to improve the rheological properties of drilling fluids at ultra-high temperatures in deep or ultra-deep drilling because of their higher thermal stability. There have been no studies on the effects of 1-ethyl-3-methylimidazolium bis(trifluoromethylsulfonyl)imide (ILA) and N-methyl, butylpyrrolidinium bis(trifluoromethylsulfonyl)imide (ILC) on the rheological properties of drilling fluids at ultra-high temperatures. This study investigates the effects of 1-ethyl-3-methylimidazolium bis(trifluoromethylsulfonyl)imide (ILA), 1-ethyl-3-methylimidazolium tetrafluoroborate (ILB), and N-methyl, butylpyrrolidinium bis(trifluoromethylsulfonyl)imide (ILC) on the rheological properties of drilling fluids at ultra-high temperatures. It compares the effects of two ionic liquids with different cation and two different ionic liquids with different anion on the rheological properties and fluid loss of water-based drilling fluids under ultra-high temperatures. The rheological model of water-based drilling fluids formulated with ILs was fitted, and the mechanisms were analyzed through TGA, XRD and contact angle experiments. 

## 2. Materials and Methods

### 2.1. Materials

**Figure 1:**
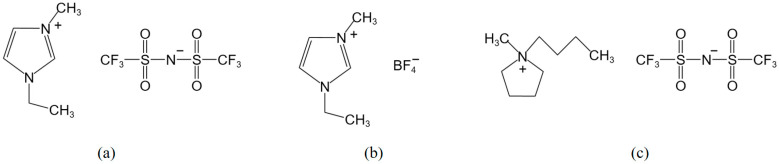
Structural formulas of three ionic liquids. (**a**) ILA; (**b**) ILB; (**c**) ILC.

The molecular structure of the following ionic liquids are shown in Figure 1: 1-ethyl-3-methylimidazolium bis(trifluoromethanesulfonyl)imide salt (ILA), 1-ethyl-3-methylimidazolium tetrafluoroborate (ILB), and N-methyl,butylpyrrolidinium bis(trifluoromethanesulfonyl)imide salt (ILC), Shanghai Chengjie Chemistry Co., Ltd. (Shanghai, China), with 99% purity.

Sodium montmorillonite (Na-Mt) was provided by Henan Longxiang Petroleum Auxiliary Co., Ltd. (Zhoukou, China), with a cation exchange capacity (CEC) of 90 mmol/100 g; polyacrylamide (PAM) and all other agents were provided by Chemical Co., Ltd., China (Beijing, China).

### 2.2. Experimental Methods

(1) Preparation of drilling fluid

According to the API standard [34], 4% sodium montmorillonite and 0.14% anhydrous sodium carbonate in mass fraction were added into 10 L of distilled water, and then the dispersion was hydrated for 24 h at room temperature to obtain a freshwater base slurry. The polymer-based slurry was obtained by adding 0.3% PAM to the freshwater-based slurry and then hydrated for 16 h.

(2) Performance testing

Rheological parameters were measured according to the API standard (API RP 13I, 2004) [35]. Different mass fractions of ionic liquids were added to the base slurry and stirred at high speed for 20 min to obtain the test specimens; the rheological parameters and filtration loss of the specimens were tested at ambient and high temperature after aging (roller oven aging for 16 h); the rheological parameters were measured with a six-speed rotational viscometer (ZNN-D6, Qingdao, China, Haitongda). Apparent viscosity (AV), yield point (YP) and plastic viscosity (PV) were calculated according to Equations (1) to (3), respectively.

(1) $$AV=0.5\theta_{600}(mPa\cdot s)$$

(2) $$PV=\theta_{600}-\theta_{300}(mPa\cdot s)$$

(3) $$YP=0.5(\theta_{300}-PV)\;(Pa)$$

where θ_600_ and θ_300_ are the measured values when the shear rate is 600 r/min and 300 r/min, respectively. Filtration loss was tested according to the API (API RP 13B-1, 2009) standard [34]. The test for API static filtration loss was accomplished with a medium-pressure filtration-loss meter with a control pressure source of 0.69 MPa.

(3) Mechanism analysis

Sodium montmorillonite (Na-Mt) is the main component of water-based drilling fluid. The montmorillonite is adsorbed by surface adsorption or interlayer adsorption of the addition, which changes the properties of the drilling fluid. Cations of ionic liquids can undergo cation exchange with the sodium of montmorillonite. Different concentrations of ionic liquids were added to the suspension of Na-Mt by mixing and shaking in a water bath for 24 h, then centrifuged and filtered, and the samples obtained were dried in an oven at 105 °C.

Na-Mt and modified Na-Mt were subjected to a thermogravimetric analysis (TGA) test using an SDT Q600 instrument (TA Instruments, Inc., New Castle, DE, USA) in air at a heating rate of 10 °C/min from room temperature to 600 °C. 

The layer spacing of Na-Mt and modified Na-Mt was obtained in the 2θ range using an X-ray diffractometer (XRD) (Ultima 5, Rigaku Co., Tokyo, Japan) at a scanning rate of 2°/min under Cu-Kα radiation at 40 kV.

The contact angle test was performed according to the literature using a JC 200D [36].

## 3. Results and Discussion

### 3.1. Effect of ILs with Different Anionic Structures on the Rheological Properties of Polymer Slurries

Rheological property is one of the most important properties of drilling fluids, which play an important role in carrying drilling cuttings and cleaning the borehole. In order to study the effect of ionic liquids with different structures on the rheological properties of water-based drilling fluids under high temperatures, firstly, ILA and ILB, which have the same imidazole cationic structure, while the anionic structures are bis(trifluoromethanesulfonimide) and tetrafluoroborate, respectively, were selected. Polymer slurries containing different concentrations of ILA and ILB were prepared and tested for their rheological properties after aging at ultra-high temperatures, and the experimental results are shown in Table 1 and Table 2.

Yield point and plastic viscosity are important parameters of drilling fluid rheology. The ratio (YP/PV) is used to measure the shear dilution of drilling fluids; the larger its value, the better the shear dilution. Generally, the value is considered to be more appropriate at 0.36–0.48, and the value is too small to effectively carry rock and clean the borehole [7,8,37]. It can be seen from Table 1 and Table 2 that for the polymer matrix without the addition of ionic liquids, the ratio of YP/PV decreases greatly after aging at 200 °C and 240 °C. The higher the temperature, the lower the value—it is almost zero, which indicates that the polymer matrix without the addition of ionic liquid has lost its rock-carrying and borehole-cleaning functions at 200 °C and 240 °C. The YP and PV ratio of the polymer slurry formulated with different concentrations of ILA and ILB increased significantly. When the concentration of ILA was 0.05%, the YP/PV ratio was 0.58 and 0.33 after aging at 200 °C and 240 °C, respectively, and the YP/PV ratio of fluids with ILB at the same concentration and high temperatures was 0.5 and 0.21, respectively. These results indicate that compared with the original polymer slurry, both ILA and ILB can improve the rheological properties of polymer slurries at ultra-high temperatures.

**Figure 2:**
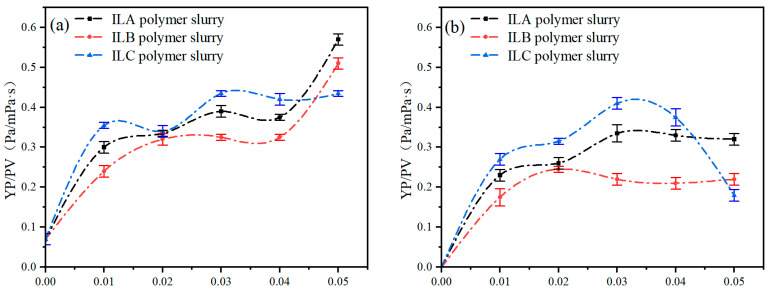
YP/PV ratio of polymer matrix with different concentrations of ILs at different aging temperatures: (**a**) 200 °C; (**b**) 240 °C.

Figure 2 shows the changes in the YP/PV ratio of polymer slurries with different concentrations of ionic liquids at different aging temperatures. As can be seen from Figure 2, the YP/PV ratio basically increased with the increase in IL concentration at different aging temperatures, but the YP/PV ratio decreased greatly with the increase in temperature. The YP/PV ratio of the polymer matrix formulated with ILA is slightly higher than that of fluids with the same concentration of ILB, indicating that for ILs with the same imidazolium cation structure, the ILA with the bis(trifluoromethanesulfonyl)amide anion has a better ability to improve rheological properties of the polymer matrix than the ILB with the tetrafluoroboric anion. Previous studies have shown that the imidazolium cation of ILA improves the high-temperature rheological properties of water-based drilling fluids by the interlayer exchange of cationic ions with sodium montmorillonite [7]. This suggests that the rheological properties of polymer slurries are not only affected by the cationic structure of ILs but also by the anionic structure of ILs. ILA has six C-F bonds and exhibits hydrophobicity, and it is less water-soluble than ILB with tetrafluoroborate anions. But ILA has four S=O groups, which can form more hydrogen bonding with water molecules or montmorillonite in the matrix, resulting in a more stable spatial network structure between the clay particles, and at higher temperatures, the ILA may be more stable and have a better ability to improve the high-temperature rheological properties of the polymer matrix.

### 3.2. Effect of ILs with Different Cationic Structures on the Rheological Properties of Polymer Slurries

In order to study the effect of different cationic structures of ILs on the rheological properties of polymer matrix, polymer matrices with different concentrations of ILC were prepared and tested for their rheological properties at ultra-high temperature aging. ILC with the anionic structure of bis(trifluoromethanesulfonyl)imide is the same as the anionic structure of ILA, but the cationic structure of ILC is pyrrolidinium cation, and the cationic structure of ILA is imidazole cation. The results are shown in Table 3. The effect of ILC on the rheological properties of polymer matrix is basically the same as that of fluids formulated with ILA and ILB. When the concentration of ILC is 0.03%, the YP/PV ratio of the polymer slurries are 0.43 and 0.42 after aging at 200 °C and 240 °C, respectively, which are slightly higher than those of the polymer slurries with the same concentrations of ILA and ILB, indicating that ILC also can improve the rheological properties of polymer matrices at ultra-high temperatures.

It can also be seen from Figure 2 that below the 0.03% concentration, the YP/PV ratio of the polymer slurry with the same concentration of ILC is slightly higher than that of fluids with ILA, while the YP/PV ratio of the polymer slurry with the same concentration of ILA is slightly higher than that of fluids with ILB. This suggests that the anionic and cationic structures both affect the thermal stability of ILs. For ILA and ILB with the same imidazolium cation, the thermal stability of ILA with bis(trifluoromethanesulfonyl)imide anion is higher than that of ILB with tetrafluoroborate cation, and for ILA and ILC with the same anion bis(trifluoromethanesulfonyl)imide, the thermal stability of ILC with pyrrolidinium cation is higher than that of ILB with imidazolium cation, which affects the high-temperature rheological properties of water-based drilling fluids. 

Reasonable rheological parameters of drilling fluid can effectively improve drilling efficiency. The drilling fluid rheology model is crucial for the accurate calculation of rheological parameters and drilling hydraulic parameters. Drilling fluids are usually non-Newtonian fluids, and their rheological properties are described by mathematical models. Although the Bingham model is the most commonly used rheological model, the Power law and Herschel–Bulkley models are currently considered closer to practical applications [38]. 

**Figure 3:**
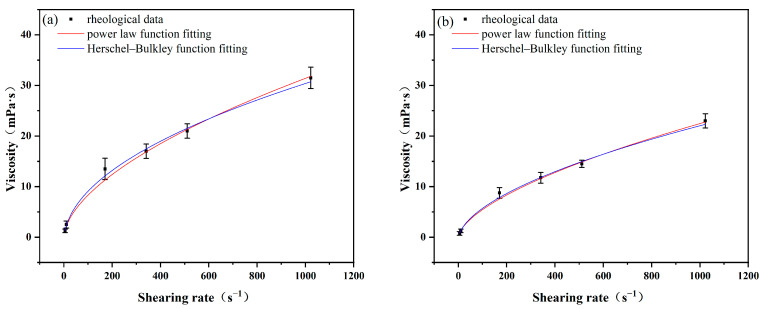
Fitting of rheological data of ILA at different temperatures: (**a**) 200 °C; (**b**) 240 °C.

**Figure 4:**
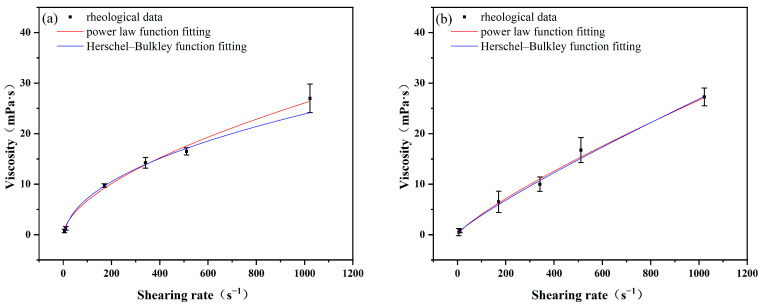
Fitting of rheological data of ILC at different temperatures: (**a**) 200 °C; (**b**) 240 °C.

Based on the rheological data of polymer matrices formulated with ILA and ILC, the rheological model was fitted using Origin 2021pro. The model parameters and correlation coefficients (R^2^) in the corresponding rheological model equations can be obtained by the equations of the two rheological models. The values of the model coefficients k (consistency index) and n (fluidity index) for the Power law model and the Herschel–Bulkley model are shown in Table 4 and Figure 3 and Figure 4.

Power law: $\tau =k\cdot \gamma^{n}$, where *k* is the consistency index in Pa, and *n* is the fluidity index, dimensionless.

Herschel–Bulkley: $\tau =k\cdot \gamma^{n}+\tau_{y}$, where *k* is the consistency index in Pa, *n* is the fluidity index, dimensionless, and *τ_y_* is the minimum shear stress required for fluid flow in Pa·s.

From Table 4 and Figure 4, it can be seen that the R^2^ values fitted with the two rheological models are 0.9961 for polymer matrix formulated with ILA after aging at 200 °C, and for the polymer matrix formulated with ILC, the R^2^ values fitted with the two rheological models are 0.9958 and 0.9975, respectively. The R^2^ obtained from the Herschel–Bulkley model is slightly higher than that from the Power law model at this temperature. The higher the R^2^ value, the better the agreement between experimental values and model predictions, which makes Herschel–Bulkley a more suitable model. The R^2^ values fitted with the two rheological models are equal at 240 °C; both models can give better predictability. 

It can also be seen that the k value of polymer matrices with ILs decreases with an increase in temperature, while the n value increases with an increase in temperature. This implies that the enhancing effect of ILs on the rheological ability of the polymer slurries decreases with increasing temperature, which is consistent with the conclusions of the previous analysis.

### 3.3. Influence of ILs on the Filtration Loss of Polymer Slurries

**Figure 5:**
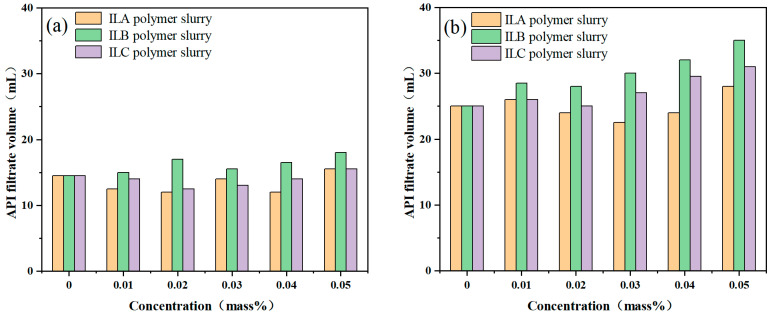
The filtration loss of polymer slurries with different ILs after aging at different temperatures: (**a**) 200 °C; (**b**) 240 °C.

The filtration-loss characteristics of drilling fluids determine the ability to form mud cakes in the well wall, which directly affect the stability of the well wall. At high temperatures, the filtration-loss volume of drilling fluids increases significantly, and the penetration of drilling fluids into the formation, which leads to the hydration and expansion of clay minerals in the formation, results in the destabilization of the well wall. Polymer slurries containing different concentrations of ILA, ILB and ILC were prepared, and the API filtration loss was tested after aging at ultra-high temperatures. The experimental results are shown in Figure 5.

It can be seen from Figure 5 that the filtration loss increases greatly with increasing temperature, but none of the three ionic liquids can reduce the ultra-high temperature filtration loss of the polymer slurries. However, among the three ionic liquids, a low concentration of ILA can slightly reduce the API filtration loss of polymer matrices after ultra-high temperature aging. One of the methods to control the volume of filtration loss is to improve the viscosity of the filter liquid. The ILs mainly improve the ratio of YP/PV but not the viscosity of the drilling fluids, which is also reported in other literature [7,31]. 

### 3.4. TGA

**Figure 6:**
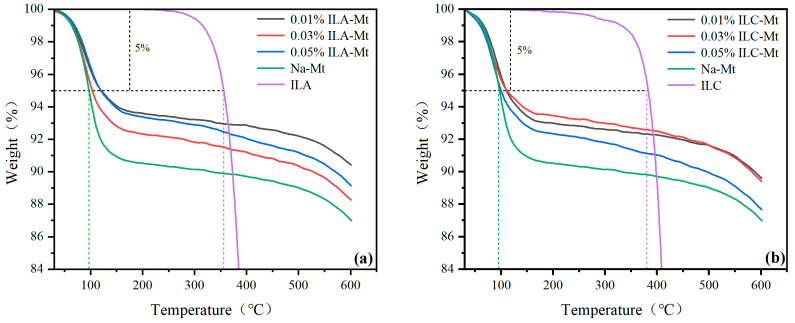
TGA curves of Na-Mt modified by different ILs: (**a**) ILA; (**b**) ILC.

The literature has reported that the thermal stabilities of 1-ethyl-3-methylimidazolium bis(trifluoromethanesulfonimide) salt (ILA) and N-methyl, butylpyrrolidinium bis(trifluoromethanesulfonimide) salt (ILC) are higher than that of 1-ethyl-3-methylimidazolium tetrafluoroborate (ILB); the latter is thermally decomposed at 330 °C [32,33]. The TGA curves of unmodified sodium montmorillonite (Na-Mt) and Na-Mt modified with different concentrations of ILA and ILC are shown in Figure 6a,b. The mass loss of unmodified Na-Mt occurred at 100 °C, 135 °C and 450 °C, respectively. These mass losses were attributed to the dehydration of the free water, the Na-Mt hydrated cations and the montmorillonite structural water, respectively. Na-Mt modified by ILA and ILC exhibited less mass loss at 100 °C, suggesting that the Na-Mt interlayer water was replaced by the cations of ILA and ILC.

It can be seen from Figure 6a that the thermal decomposition temperature of ILA is about 350 °C, and the Na-Mt modified with 0.05%, 0.03% and 0.01% ILA has less weight loss compared with the unmodified Na-Mt, which indicates that the thermal stability of Na-Mt is improved by the modification of ILA, and at the same time, the weight loss of the Na-Mt modified with low concentration (0.01%) is less than that of the Na-Mt modified with high concentration (0.05%), which indicates that the thermal stability of Na-Mt modified with low concentration ILA is slightly higher than that of Na-Mt modified with high concentration ILA. Chemical cation-exchange monolayer adsorption occurs at lower concentrations, while multilayer physical adsorption occurs at higher concentrations; the former is more stable compared to the latter [39,40,41]. The thermal decomposition temperature of ILC is about 380 °C, which is higher than that of ILA. Na-Mt modified with ILC exhibited a higher thermal decomposition temperature. Overall, the addition of low concentrations of ILA and ILC effectively improved the thermal stability of Na-Mt. Because the thermal stability of bis(trifluoromethanesulfonimide) anionic structures of ILs is higher than those of other reported ILs, and the decomposed temperature is over 350 °C, the PAM slurry formulated with ILA or ILC can maintain better rheological properties.

### 3.5. XRD

**Figure 7:**
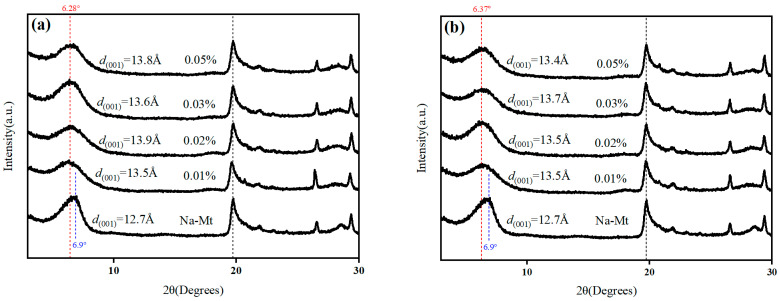
XRD plots of Na-Mt modified with different ILs: (**a**) ILA; (**b**) ILC.

XRD results of Na-Mt modified with different concentrations of ILA and ILC are shown in Figure 7. The layer spacing of the blank Na-Mt is 12.7 Å, which is consistent with that reported in the literature [39,41]. With the increase in ILA concentration, the layer spacing of modified Na-Mt gradually enlarged and reached a maximum of 13.9 Å at the ILA concentration of 0.02%, and then the layer spacing of Na-Mt gradually decreased with the further enlargement of the ILA concentration, but it was still larger than that of the blank Na-Mt; the layer spacing of modified Na-Mt with ILs increased compared with that of the original Na-Mt. The spacing layers of Na-Mt were enlarged because the cations of the ionic liquids were intercalated into the Na-Mt interlayer. It has been reported that the arrangement of cations in the Na-Mt interlayer is related to its type and concentration. The cation interlayer exchange adsorption is a dynamic adsorption; when the concentration of ionic liquids is low, the cations in the Na-Mt interlayer may be in a single-molecule vertical state, and with the increase in IL concentration, the cation changes to be in the bimolecular layer lying arrangement, which leads to reducing the layer spacing [42].

### 3.6. Contact Angle

**Figure 8:**
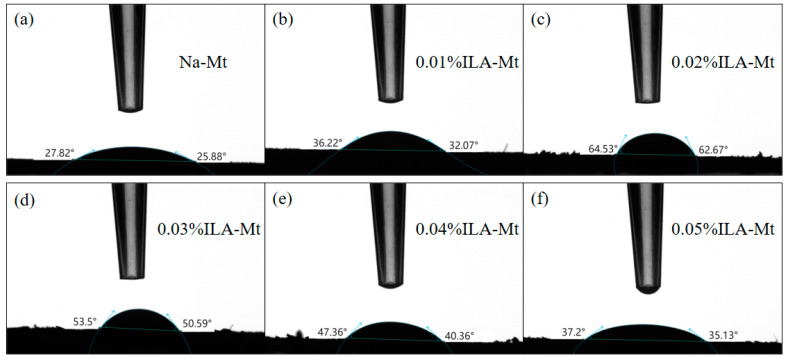
Contact angle test of Na-Mt modified with different concentrations of ILA: (**a**) Na-Mt; (**b**) 0.01%ILA-Mt; (**c**) 0.02%ILA-Mt; (**d**) 0.03%ILA-Mt; (**e**) 0.04%ILA-Mt; (**f**) 0.05%ILA-Mt.

**Figure 9:**
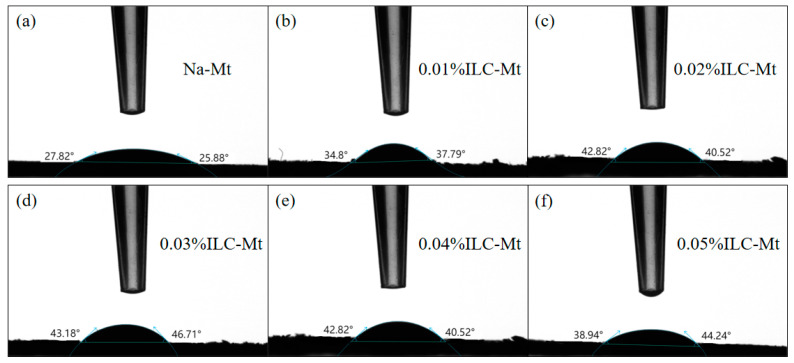
Contact angle test of Na-Mt modified with different concentrations of ILC: (**a**) Na-Mt; (**b**) 0.01%ILC-Mt; (**c**) 0.02%ILC-Mt; (**d**) 0.03%ILC-Mt; (**e**) 0.04%ILC-Mt; (**f**) 0.05%ILC-Mt.

The contact angle tests of the original Na-Mt and the Na-Mt modified with different concentrations of ILA and ILC are shown in Figure 8 and Figure 9. The contact angle of the initial Na-Mt was 25.9°–27.8°, which is in general agreement with the literature [42]. The contact angle reached a maximum value of 62.8°–64.5° when the concentration of ILA was increased from 0.01% to 0.02%, but the contact angle of the modified Na-Mt gradually decreased with the further increase in the concentration, and when the concentration was increased from 0.02% to 0.05%, the contact angle showed a small decrease, but the contact angle compared to that of the original Na-Mt still increased significantly. The Na-Mt modified with ILA possesses a larger contact angle compared to that modified with the ILC. The maximum value of the Na-Mt modified with 0.02% ILA was 64.53°, while the Na-Mt modified with ILC was 46.71°, which may be due to the different cation. 

## 4. Mechanism Analysis

**Figure 10:**
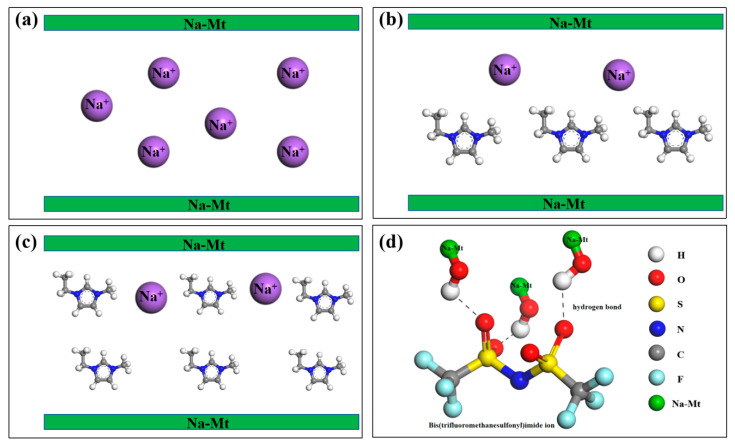
Schematic diagram of the mechanism of ionic liquid interaction with sodium montmorillonite: (**a**) layered Na-Mt; (**b**) monomolecular layer adsorption at lower concentrations; (**c**) bimolecular layer adsorption at higher concentrations; (**d**) hydrogen bonding between Na-Mt and the ionic liquid.

Experiments have showed that low concentrations of ILA, ILB and ILC can effectively improve the rheological properties of water-based drilling fluids at ultra-high temperatures, which may be due to the strong interactions established between the ionic liquids and the water-based drilling fluids system, which include (in the case of ILA): (1) Na-Mt is composed of a layer structure with negatively charged (Figure 10a), and the layered Na-Mt may be stripped at higher temperatures, which results in more dispersion and an increase in viscosity; (2) the cationic1-ethyl-3-methylimidazole portion of ILA enters into the Na-Mt interlayer through cation exchange intercalation and changes the interlayer spacing, which may be monomolecular layer adsorption at lower concentrations (Figure 10b) and bimolecular layer adsorption at higher concentrations (Figure 10c), thus improving the thermal stability of modified Na-Mt; and (3) possible hydrogen bonding between the hydroxyl groups in the Na-Mt and the trifluorosulfonimide anion of the ionic liquid (Figure 10d). Through these effects, it is easier to connect end faces and layer faces of Na-Mt at ultra-high temperatures, which, to some extent, enhances the structural stability between Na-Mts and inhibits ultra-high temperature dispersion or agglomeration of Na-Mts, thus improving the rheological properties of drilling fluids at ultra-high temperatures.

## 5. Conclusions

The following conclusions can be drawn from the above experiments:

(1) Compared with the PAM slurry without ionic liquids, the YP/PV ratio of PAM slurry formulated with the three ionic liquids at low concentrations increased significantly after aging at ultra-high temperatures of 200 °C and 240 °C. ILA and ILB with different anion structures can improve the ultra-high temperature rheological properties of aqueous drilling fluids, and ILA and ILC with different cation structures can also improve the ultra-high temperature rheological properties of aqueous drilling fluids.
(2) The TG thermal stability of N-methyl, butylpyrrolidinium bis(trifluoromethanesulfonimide) (ILC) is higher than that of 1-ethyl-3-methylimidazolium bis(trifluoromethanesulfonimide) (ILA), and that of 1-ethyl-3-methylimidazolium tetrafluoroborate (ILA) is higher than that of 1-ethyl-3-methylimidazolium tetrafluoroborate (ILB), which is consistent with the fact that the three ionic liquids improve the YP/PV ratio of drilling fluids under ultra-high temperatures. Both the Herschel–Bulkley model and the Power law model can be fitted with the rheological model of PAM slurries formulated with ILA and ILC.
(3) The cationic interlayer exchange between the organic cations of ionic liquids and sodium montmorillonite can enhance the ultra-high temperature stability of sodium montmorillonite and improve the rheological properties of aqueous drilling fluids; the s=o bond in bis(trifluorosulfonyl)imide ions and the hydroxyl group in sodium montmorillonite may form hydrogen bonds to improve the rheology of water-based drilling fluids.
(4) None of the three ionic liquids—ILA, ILB and ILC—can reduce the ultra-high temperature filtration loss of PAM-based drilling fluids.
(5) ILA, ILB and ILC may be used to improve the rheological properties of drilling fluids at ultra-high temperatures in deep or ultra-deep drilling because of the higher thermal stability they provide.

## Figures and Tables

**Table 1:** Rheological properties of polymer slurries with different concentrations of ILA.

Concentration of ILA	Experimental Conditions	AV (mPa·s)	PV (mPa·s)	YP (Pa)	YP/PV (Pa/mPa·s)
0	room temperature	22.5	12.5	10	0.8
	200 °C	6.5	6	0.5	0.08
	240 °C	6.5	6.5	0	0
0.01%	room temperature	18.75	10.5	8.25	0.79
	200 °C	5.25	4	1.25	0.31
	240 °C	5.5	4.5	1.0	0.22
0.02%	room temperature	19	10	9.0	0.90
	200 °C	6.0	4.0	1.5	0.33
	240 °C	8.25	6.5	1.75	0.27
0.03%	room temperature	20	12	8	0.67
	200 °C	7.0	5.0	2.0	0.40
	240 °C	13.5	10.0	3.5	0.35
0.04%	room temperature	18	11	7	0.64
	200 °C	11.0	8.0	3.0	0.38
	240 °C	12.5	9.5	3.0	0.32
0.05%	room temperature	19	11	8	0.73
	200 °C	19	12	7	0.58
	240 °C	12	9	3	0.33

**Table 2:** Rheological properties of polymer slurries with different concentrations of ILB.

Concentration of ILB	Experimental Conditions	AV (mPa·s)	PV (mPa·s)	YP (Pa)	YP/PV (Pa/mPa·s)
0	room temperature	22.5	12.5	10	0.80
	200 °C	6.5	6	0.5	0.08
	240 °C	6.5	6.5	0	0
0.01%	room temperature	23.5	14	9.5	0.68
	200 °C	5	4	1	0.25
	240 °C	4.75	4.0	0.75	0.19
0.02%	room temperature	20.5	11.5	9	0.78
	200 °C	8.5	6.5	2.0	0.31
	240 °C	5	4	1	0.25
0.03%	room temperature	22	14.5	7.5	0.52
	200 °C	8	6	2	0.33
	240 °C	6.75	5.5	1.25	0.23
0.04%	room temperature	20	10.5	9.5	0.90
	200 °C	4	3	1	0.33
	240 °C	6	5	1	0.2
0.05%	room temperature	20.0	10	10	1.0
	200 °C	3	2	1	0.5
	240 °C	7.25	6	1.25	0.21

**Table 3:** Rheological properties of polymer slurries with different concentrations of ILC.

Concentration of ILC	Experimental Conditions	AV (mPa·s)	PV (mPa·s)	YP (Pa)	YP/PV (Pa/mPa·s)
0	room temperature	22.5	12.5	10	0.80
	200 °C	6.5	6	0.5	0.08
	240 °C	6.5	6.5	0	0
0.01%	room temperature	21	12	9	0.75
	200 °C	4.75	3.5	1.25	0.36
	240 °C	5.75	4.5	1.25	0.28
0.02%	room temperature	18	11	7	0.64
	200 °C	6.75	5.0	1.75	0.35
	240 °C	13.75	10.5	3.25	0.31
0.03%	room temperature	19	13	6	0.46
	200 °C	14.25	10	4.25	0.43
	240 °C	8.5	6	2.5	0.42
0.04%	room temperature	17	11.5	5.5	0.48
	200 °C	14.25	10	4.25	0.43
	240 °C	12.5	9	3.5	0.39
0.05%	room temperature	19.5	12	7.5	0.63
	200 °C	23	16	7	0.44
	240 °C	10.5	9	1.5	0.17

**Table 4:** Rheological modeling constants and correlation coefficients for polymer slurries at different temperatures.

Temperature	Rheology Model	Ratio	ILA-PAM	ILC-PAM
200 °C	Power law	k	1.3640	0.7929
		n	0.5184	0.5428
		R^2^	0.9961	0.9958
	Herschel–Bulkley	k	1.2630	0.5361
		n	0.5294	0.5994
		$\tau_{y}$	0.3168	0.9840
		R^2^	0.9961	0.9975
240 °C	Power law	k	0.6542	0.2419
		n	0.5602	0.7468
		R^2^	0.9945	0.9916
	Herschel–Bulkley	k	0.6039	0.2414
		n	0.5717	0.7471
		$\tau_{y}$	0.1918	0.0054
		R^2^	0.9945	0.9916

## Data Availability

Data are contained within the article.
